# Supplementary material for: Identification of QTLs Controlling Resistance to Anthracnose Disease in Water Yam (Dioscorea alata)
Source: Genes (Basel). 2022 Feb 14;13(2):347. doi: 10.3390/genes13020347 (PMC8872494; doi:10.3390/genes13020347)
Supplement: Supplementary file 1 [file genes-13-00347-s001.zip › genes-1580193-supplementary.pdf]

Supplementary Table and Figures

Table S1. List of the putative genes identified in the vicinity of the QTL associated with yam anthracnose disease (YAD).

Trait	Chr	Gene ID	Mapping interval	Putative function
YAD	7	DRNTG_08663.1*	10.596 to 9.217	Plant defense mechanism / disease resistance (GDSL-like Lipase/Acylhydrolase)
		DRNTG_08664.1*		Plant defense mechanism / regulating the resistance against bacterial blight (Protein kinase domain)
		DRNTG_23336.1*		Plant defense mechanism / regulating the resistance against bacterial bligh (Protein kinase domain)
		DRNTG_23346.1		Plant growth
	15	DRNTG_14305.1*	28.817 to 30.900	N-terminal α/β domain gene involve in fungal disease resistance
		DRNTG_14560.1		Regulator of growth and development
	18	DRNTG_18245.1*	61.345 to 61.432	ANTH domain Putative clathrin assembly protein
		DRNTG_29617.1*		WD domain - WD40 repeat-containing protein
		DRNTG_31006.1		Biosynthesis of hormones

The genes with * represent the genes involve in plant mechanism

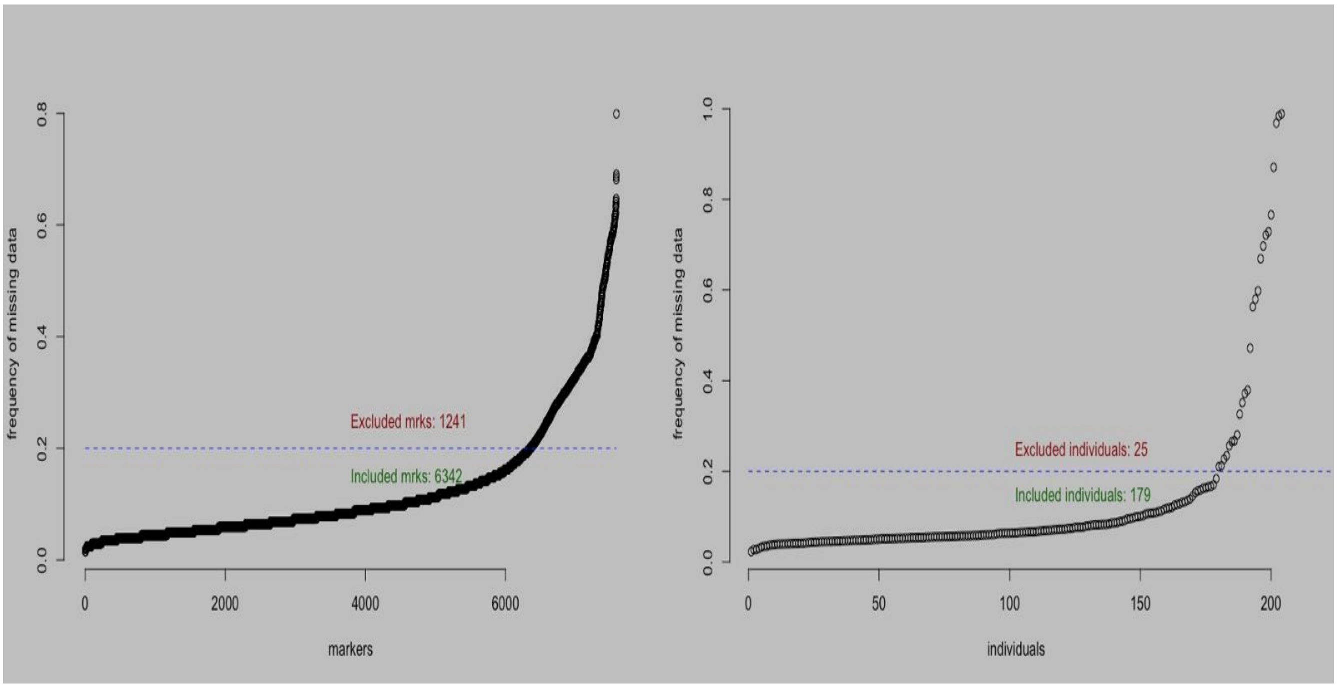

Figure S1. SNP quality assessment for missing markers and genotypes (Missing threshold = 20%) showing informative markers (the left figure) and genotypes with good coverage (right figure) for linkage map construction. The blue horizontal dotted lines represent the threshold.

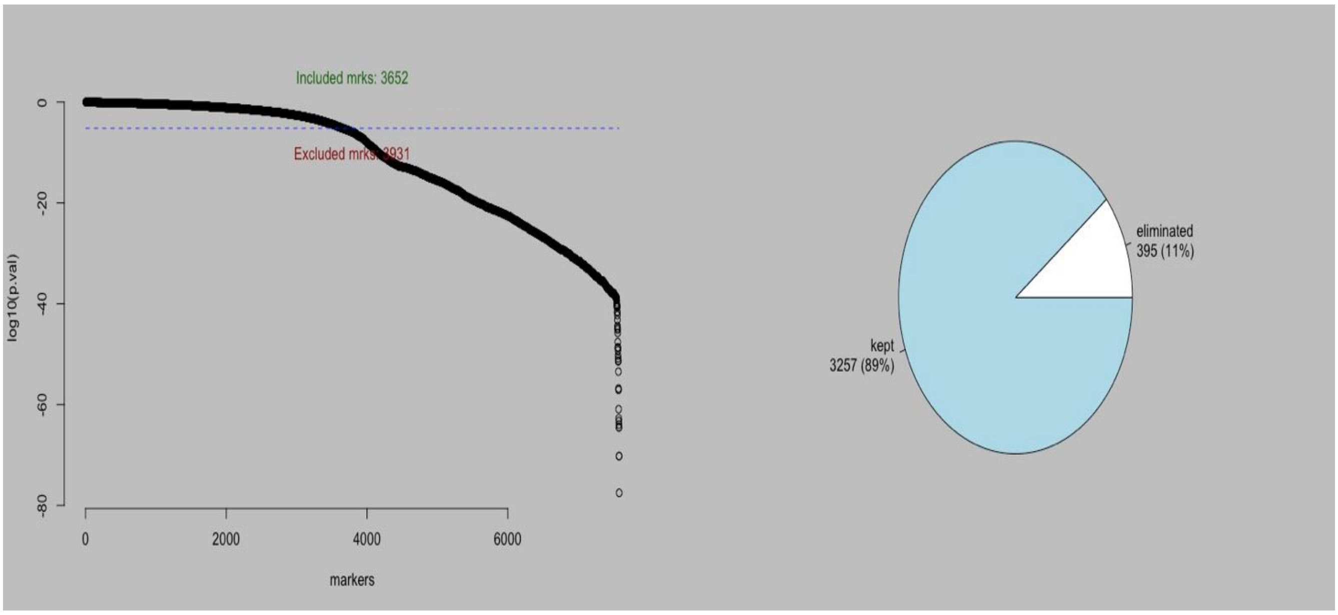

Figure S2. Evaluation of polymorphic SNP markers in the biparental population for segregation distortion using chi- square test.

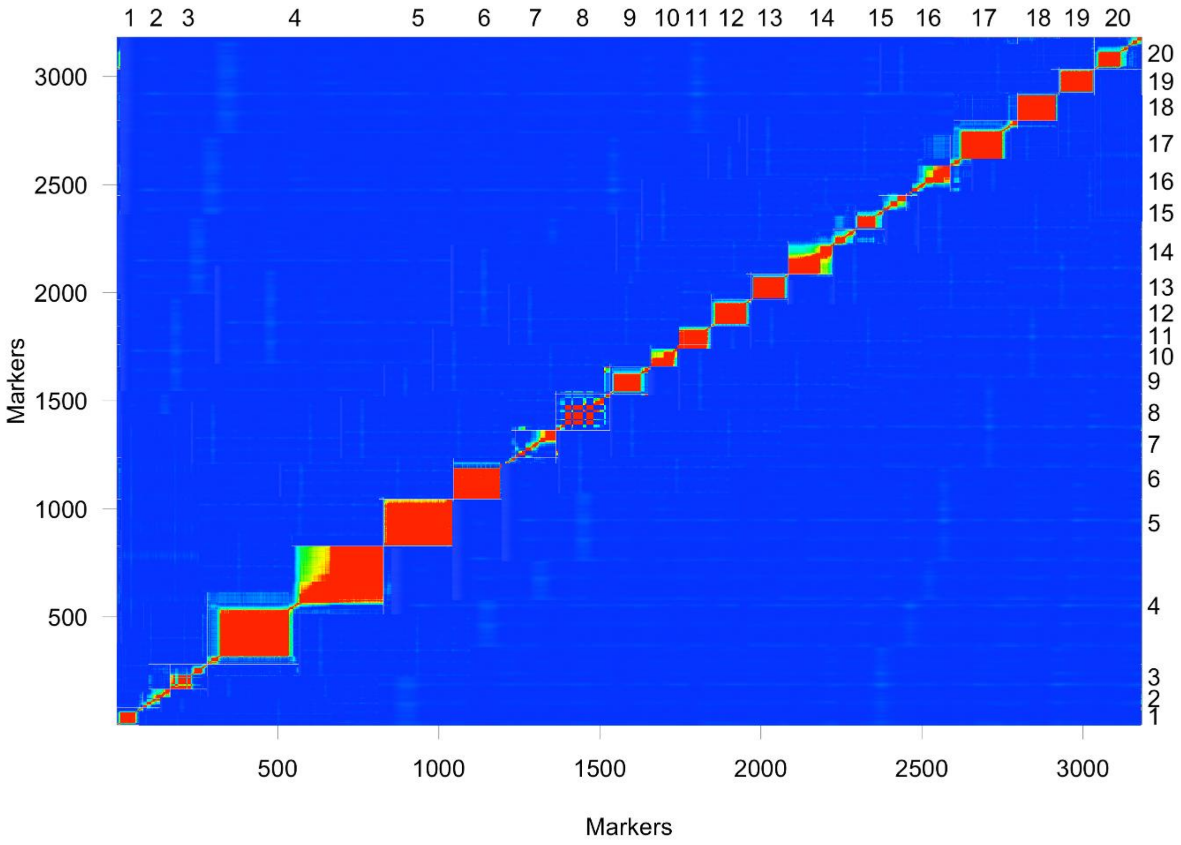

Figure S3. Pairwise recombination fractions and LOD scores of SNP markers used in the study. A pairwise comparison of recombination fractions after SNP markers were ordered across the 20 chromosomes. Recombination fractions are in the upper left triangle, the LOD scores are in the lower right triangle and the red corresponds to the large LOD or a small recombination fraction.

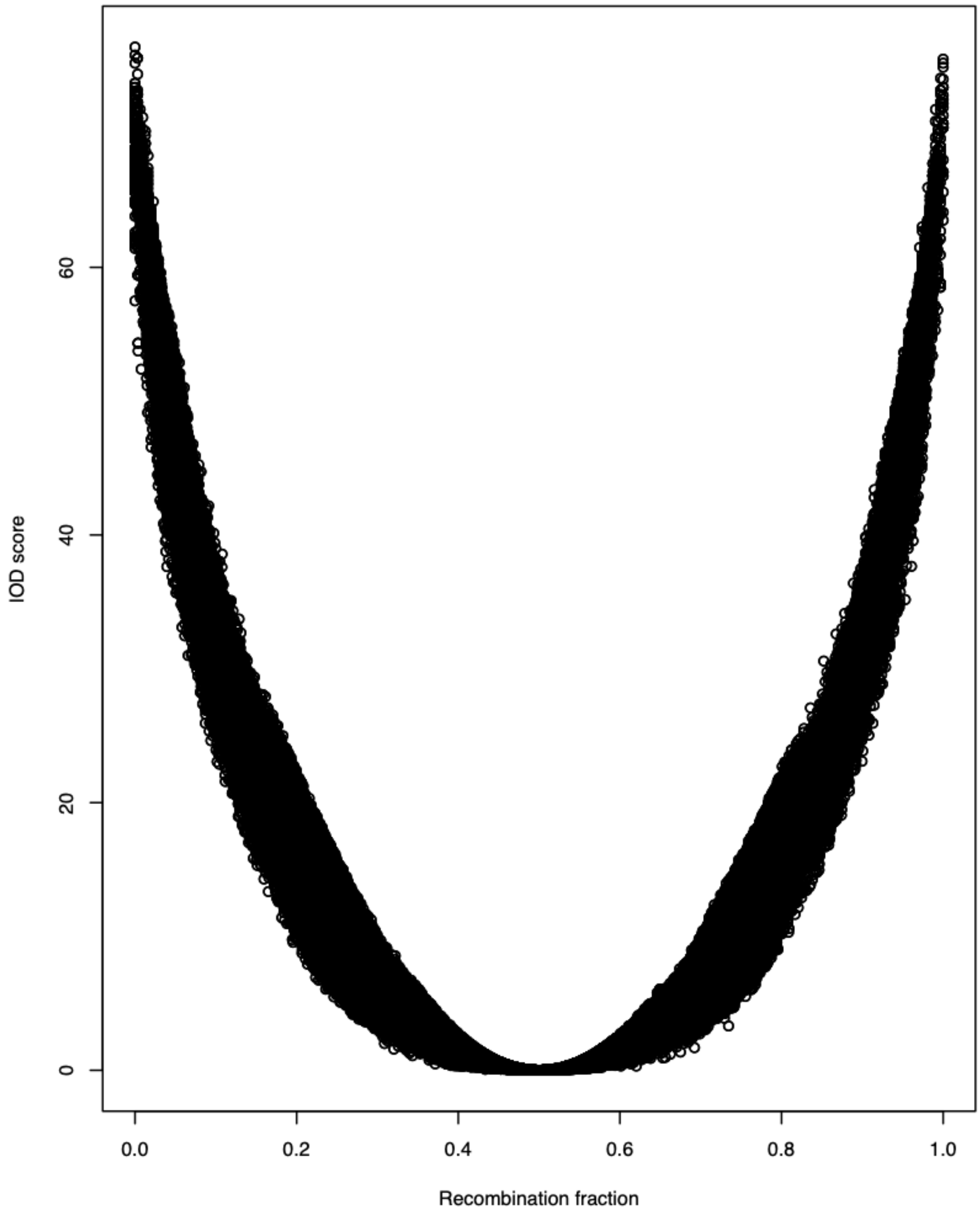

Figure S4. Plot of recombination fraction against the LOD score showing perfect alignment as no half circles were observed.
